# Residual effects of eszopiclone and placebo in healthy elderly subjects: a randomized double-blind study

**DOI:** 10.1007/s41105-017-0101-2

**Published:** 2017-05-19

**Authors:** Jun Takahashi, Takashi Kanbayashi, Sachiko Ito Uemura, Youhei Sagawa, Kou Tsutsui, Yuya Takahashi, Yuki Omori, Aya Imanishi, Masahiro Takeshima, Masahiro Satake, Tetsuo Shimizu

**Affiliations:** 1 Department of Psychiatry, Akita University Graduate School of Medicine, Akita, Japan; 2 International Institute for Integrative Sleep Medicine (WPI-IIIS), University of Tsukuba, Tsukuba, Japan; 3 Department of Physical Therapy, Akita University Graduate School of Health Sciences, Akita, Japan

**Keywords:** Hypnotics, Residual effects, Benzodiazepine, Critical flicker fusion test, Eszopiclone

## Abstract

Next-day residual effects are a common problem with current hypnotics. The purpose of the present study was to evaluate the residual effects of eszopiclone on the physical and cognitive functions of healthy elderly people in the early morning and the day following drug administration. Four men and six women aged 63–72 years were administered eszopiclone 1 mg or placebo in a randomized, double-blind and crossover design. Measures of objective parameters and subjective ratings were obtained at 4:00, 6:00, and every 2 h from 6:00 to 16:00 hours. For the timed up-and-go test, the main effects of time were seen. For the critical flicker fusion, eszopiclone had significantly worse results compared to placebo in early morning (4:00). There were no significant differences between eszopiclone and placebo in other objective assessments. For the sleep latency, eszopiclone had significantly shorter results compared to placebo (eszopiclone vs placebo = 28.4 vs 52.5 min, *p* = 0.047). Feeling of deep sleep and the number of wake after sleep onset did not show any significant differences between eszopiclone and placebo. Based on the above results, the changes of physical and cognitive functions in the healthy elderly after taking hypnotics, it was found that eszopiclone 1 mg is likely to be unharmful for the healthy elderly. Further studies of elderly insomniacs with midnight awakenings are needed.

## Introduction

Previous studies have reported that the prevalence of insomnia is 8–18%, and females are more prevalent than males [1, 2]. In Japan, the prevalence of insomnia is 17.3% in males and 21.5% in females [3–5]. The occurrence of insomnia symptoms appeared to be associated with advancing age.

Although national panels have long cautioned against the use of hypnotics in the management of chronic insomnia [6], the use rate of hypnotics of elderly people is about 10–15% which is about 5 times that of young adults [7] and prescription of hypnotics is gradually increasing every year [8].

Hypnotics including benzodiazepine (BZ) relatively affect the physical and cognitive functions the next day. The association between the residual effects of hypnotics and road traffic accidents is often researched in Western countries, and it has been reported that the use of benzodiazepine hypnotics increases the odds ratio of traffic accident risk up to 1.19 [9–11].

In the elderly, the sensitivity to the drugs increases and blood concentration tends to rise because metabolic capacity declines. For this reason, the association between the use of benzodiazepine hypnotics in the elderly and the risk of falls and fracture is well established, and the use of hypnotics is often discussed as a public health problem [12, 13].

Some research evaluated the effects of hypnotic on the physical and cognitive functions in healthy adults [14, 15], but research on the elderly is few [16, 17].

Uemura et al. reported a significant difference in some functions between the use of hypnotics and placebo in the healthy elderly [16]. However, there are still few studies on the use of hypnotics in the elderly, whose physical and cognitive impairments depend on aging.

There are some studies that use hypnotics in daytime, but quite few studies use hypnotics before sleep and evaluate their effects during the night [16, 17]. According to a regional survey in Japan in 2008, brotizolam, zolpidem, and rilmazafone are the top three of the most prescribed hypnotics [18]. The common feature of these hypnotics is the short half-life effects.

These short-acting hypnotics are considered to have no residual effect after 8 hours of sleep. Since, mid-arousal by urination in the elderly increases, the total sleep time tends to be short. Its evaluation for functions about 5 h after oral administration is considered to be more practical.

Eszopiclone, which is classified as a non-BZ that acts on GABA/BZ receptor chloride channel complex, is recently available in Japan. Non-BZ agents are much more selective for the BZ receptor subtypes (GABA_A_) and have reportedly fewer side effects [19, 20].

We evaluated the differences of the elderly’s physical and cognitive functions and subjective assessments using eszopiclone at night by randomized and double-blinded trials.

## Methods

### Design

The study was a randomized, double-blind, active (eszopiclone 1 mg) and placebo-controlled, two-period crossover study. Akita University Ethics Committee approved the protocol. The study was carried out in accordance with the principles based on the Declaration of Helsinki, and written informed consent was obtained from all subjects.

### Subjects

4 men and 6 women aged 63–72 years and who were all healthy were eligible to enroll if they had a usual bedtime between 20:00 and 24:00 hours. The subjects agreed to avoid exercise that was strenuous or that they were unaccustomed to during the study. They were required to be in good health, as confirmed by their medical history, physical examination, and laboratory tests (hematology and clinical biochemistry). Exclusion criteria included the use of hypnotics within the previous 1 year, a history of drug or alcohol abuse and of repeated falls or a fracture secondary to a fall within the past 2 years. Subjects were required to abstain from prescription and nonprescription drugs and supplements. During the study, subjects were required to limit their daily consumption of alcoholic beverages containing less than 30 g alcohol per day. Alcoholic beverages were not permitted from 48 h before admission to the unit or before pre-study and post-study visits. Both caffeine and nicotine were prohibited 24 h before each visit to the experimental room set up at a local hotel.

### Procedure

**Fig. 1:**
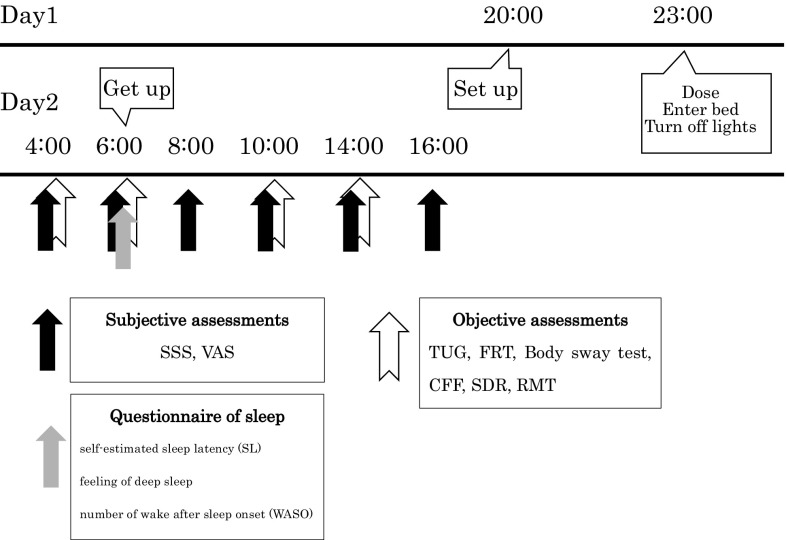
Procedure. Eszopiclone or placebo was orally given to each subject at bedtime (23:00 hours, Day 1) and lights were turned off. On the morning following dosing, lights were turned on at 4:00 and 6:00 hours (5 and 7 h post-dose) on Day 2. Measurement of subjective parameters was obtained at 4:00, 6:00, 8:00, 10:00, 14:00 hours, and 16:00 hours on Day 2. Questionnaire on sleep was obtained at 6:00 hour on Day 2. Objective parameters were obtained at 4:00, 6:00, 10:00, and 14:00 hours on Day 2. Subjects remained in the hotel from early on the evening of day 1 until 16:00 hour on day 2. *SSS* Stanford Sleepiness Scale, *VAS* visual analog scale, *Questionnaires of sleep* questionnaires of self-estimated sleep latency (SL), *feeling of deep sleep* number of wake after sleep onset (WASO), *CFF* critical flicker fusion, *FRT* functional reach test, *TUG* the timed up-and-go test, *SDR* simple discriminatory reaction, *STM* short-term memory test

Subjects were randomized to treatment sequences including eszopiclone 1 mg and placebo administered in a crossover fashion, with a 6-day washout period between each treatment. Hypnotics or placebo were orally administered to each subject at bedtime (23:00) and lights were then turned off. On the morning following dosing, lights were turned on at 4:00 and 06:00 (5 and 7 h post-dosing) (Fig. 1). Measurement of objective parameters and subjective parameters were obtained beginning at 4:00 (5 h post-dosing) on Day 2. Subjects remained in the experimental room and close to bedrooms from early in the evening until 17 h post-dosing. All subjects took the same breakfast.

### Objective assessments

#### Physical functions

##### The timed up-and-go (TUG) test

The TUG test was performed according to the method described by Podsiadlo and Richardson [21]. Participants had to stand up from a sitting position (height of chair = 40 cm) and walk 3 m along a line, perform a 180° turn, and walk back to the chair and sit down; this was timed. The TUG tests were conducted twice, and the best time (where appropriate) was used. Smaller values were better results for TUG.

##### Functional reach test (FRT)

In the FRT, the protocol described by Duncan et al. [22] was applied and a GB-200^®^ (OG giken, Okayama, Japan) was used. Each participant was positioned with one arm raised at 90° and fingers extended. A yardstick was mounted on the wall at shoulder height. The distance that a participant could reach while extending forward from the initial upright posture to the maximal anterior leaning posture without moving or lifting the feet was visually measured in cm, according to where the middle fingertip was positioned on the mounted yardstick. The distances were measured for two attempts, and these were averaged to obtain the FR score. Larger values were better results for FRT.

##### Body sway test

Body sway test (cm) reflects the standing balance using a stabilometric platform [Zebris WinFDM system (platform), Zebris Medical GmbH, Isny im Allgäu, Germany; Foot Print for Windows^®^ (software), Inter Reha, Tokyo, Japan]. Subjects stood on the platform for 30 s in bare feet and with their vision fixed at a point 2 m in front of them at eye level (eyes open) or with their eyes closed. The sum of the tracks of the center of gravity is the body sway test; the extent of movement of the center of pressure directly relates to the subject’s ability to maintain static balance. For the body sway test, smaller values were better in terms of functionality of the subject.

#### Cognitive functions

##### Critical flicker fusion (CFF) test

This test is believed to assess the integrative capacity of the central nervous system (CNS) using TAKEI KIKI KOGYO FLICER ITEM No. 501. Subjects were required to discriminate flicker from fusion, and vice versa, of four light-emitting diodes arranged in a 1 cm square on a black background. Individual thresholds were determined by the psychophysical method of limits on two ascending (flicker to fusion) and two descending (fusion to flicker) scales. The mean of these two ascending and two descending presentations was used as the threshold frequency in Hz. A decreased threshold was indicative of impairment. The test has been shown to be sensitive to psychoactive compounds [23].

##### Simple discrimination reaction (SDR) test

The SDR test is included in a Performance Test Program (NoruPro Light Systems, Inc., Tokyo, Japan). The test measures the reaction time and hand–eye coordination skills of the subjects. Subjects were required to right click on a mouse when a blue circle was lit, or left click when a white circle was lit, as quickly as possible. The mean total reaction time (s) and the rate of correct answer (%) of 60 trials were recorded. An increase (slowing) in reaction time was indicative of impairment. The test has been shown to be sensitive to psychoactive compounds [23]. Smaller values were better, in terms of functionality of the subject for the SDR reaction time, while larger values were better for the SDR accuracy rate (%).

##### Recognition Memory Test (RMT)

The RMT test is also included in the Performance Test Program (NoruPro Light Systems, Inc., Tokyo, Japan). Subjects were required to click the right mouse button when the same number that was displayed three times before the current one appeared, or click the left mouse button when a different number appeared. The rate (%) of correct answers in 60 trials was recorded. A decrease in correct answers was indicative of impairment. The test has been shown to reflect the retention of short-term memory. Prior to the study, the subjects underwent an extensive training session to preclude learning effects.

### Subjective assessments

The subjects’ sleepiness was evaluated using the Stanford Sleepiness Scale (SSS) [24]. Alertness, well-being, and fatigue were evaluated with a visual analog scale (VAS) at 4:00 hour (Day 2) and every 2 h from 4:00 to 16:00 hour (i.e., 4:00, 6:00, 8:00, 10:00, 12:00, 14:00, 16:00 hour) (Day 2). The scale’s extremes were ‘very drowsy–very alert,’ ‘very bad–very good,’ and ‘very tired–very rested.’ In terms of functionality of the subject for the SSS, smaller values were better, while larger values were better for the VAS.

In the next morning after the drug administration, the subjects were asked to fill out questionnaires of self-estimated sleep latency (SL), feeling of deep sleep, and number of wake after sleep onset (WASO).

### Safety

Physical examinations, vital sign measurements, laboratory tests to ensure safety, and 12-lead electrocardiograms (ECGs) were performed pre-study and post-study. In addition, laboratory tests to ensure safety were performed prior to dosing, and vital signs were measured during each treatment period. Subjects were monitored for adverse experiences throughout the study. For each adverse event, the investigator indicated whether or not they thought the event was drug related. This determination was made while the investigator was blinded to the treatment.

### Statistical methods

For statistical analysis, we used a two-way repeated measures ANOVA with two levels of drug (eszopiclone, placebo). After checking for interaction, we did a multiple comparison using Bonferroni for the main effects of the medicines or times. We analyzed sleep latency, feeling of deep sleep, and number of wake after sleep onset with a paired *t* test. The statistical significance level was *p* < 0.05.

## Results

**Fig. 2:**
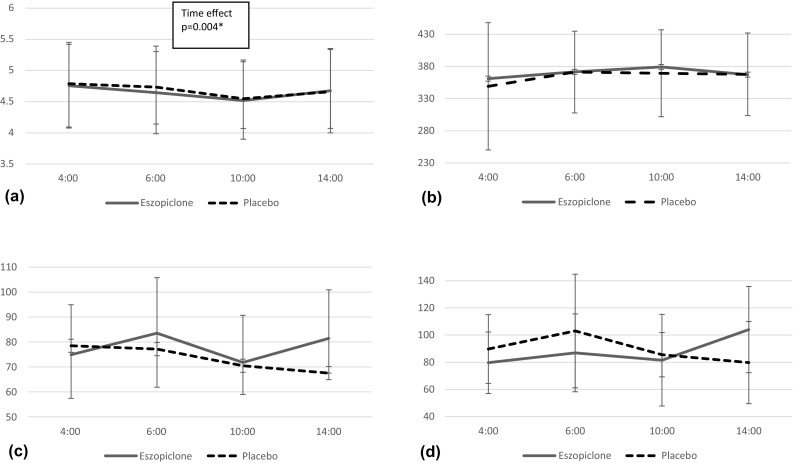
Evaluations of physical functions. For the TUG, the main effects of time were seen. There were no significant differences in FRT and Body sway test between eszopiclone and placebo. **a** TUG, the timed up-and-go test.** b** FRT, functional reach test.** c** Body sway test (eyes open).** d** Body sway test (eyes closed)

**Fig. 3:**
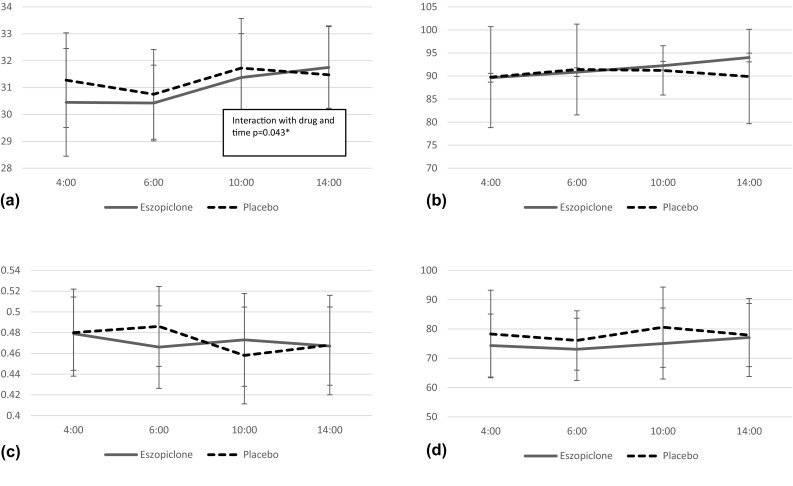
Evaluations of cognitive functions. For the CFF, eszopiclone had significantly worse results compared to placebo in the early morning (4:00). There were no significant differences in STM, SDR accuracy rate, and SDR reaction time between eszopiclone and placebo. **a** CFF, Critical Flicker Fusion (Hz). **b** SDR, Simple Discrimination Reaction Accuracy Rate (%). **C** SDR, Simple Discrimination Reaction Time (s). **d** RMT, Recognition Memory Test (%)

**Fig. 4:**
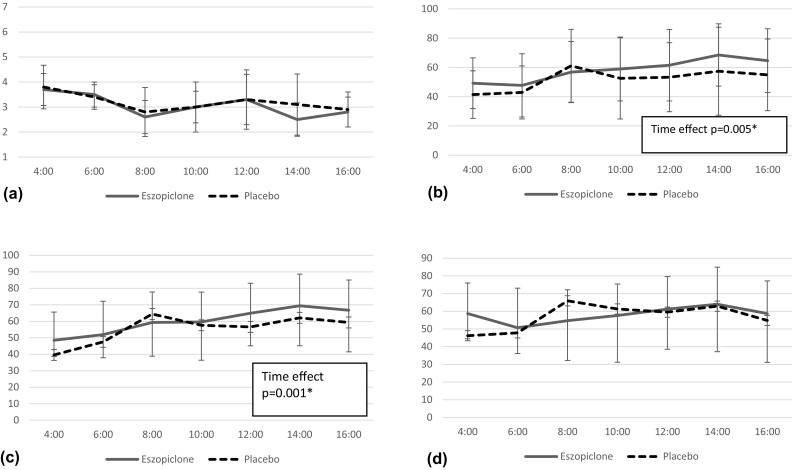
Subjective assessments. For the subjective scores of alertness and well-being using VAS, the main effects of time were seen. There were no significant differences between eszopiclone and placebo in SSS and fatigue using VAS. For the sleep latency, eszopiclone had significantly shorter results compared to placebo (eszopiclone vs placebo = 28.4 vs 52.5 min, *p* = 0.047). Feeling of deep sleep and the number of wake after sleep onset (WASO) did not show any significant differences between eszopiclone and placebo. **a** Stanford Sleepiness Scale (SSS). **b** Alertness. **c** Well-being. **d** Fatigue

During this study period, no subjects dropped out from this study procedure. The results of physical functions are shown in Fig. 2, cognitive functions in Fig. 3, and subjective assessments in Table 1 and Fig. 4.

**Table 1:** Subjective assessments of sleep parameters with eszopiclone or placebo

	Sleep parameters		
	Sleep latency (min)	Feeling of deep sleep	number of WASO
Eszopiclone (Mean ± SE)	28.4 ± 27.6	2.40 ± 1.50	1.50 ± 3.10
Placebo (mean ± SE)	52.5 ± 38.5	3.50 ± 1.26	1.80 ± 1.61
*p* value	0.047	NS	NS

For the TUG, the main effects of time were seen. For the CFF, eszopiclone had significantly worse results compared to placebo in early morning (4:00 hour). There were no significant differences between eszopiclone and placebo in other objective assessments.

For the subjective scores of alertness and well-being using VAS, the main effects of time were seen. There were no significant differences between eszopiclone and placebo in SSS and fatigue using VAS. For the sleep latency, eszopiclone had significantly shorter results compared to placebo (eszopiclone vs placebo = 28.4 vs 52.5 min, *p* = 0.047) (Table 1). There were no significant differences between eszopiclone and placebo in feeling of deep sleep and the number of wake after sleep onset (WASO).

## Discussion

Uemura et al. [16] and Ito et al. [17] examined the effects on the next day of hypnotic to healthy young people. Zolpidem and zaleplon were reported to have significantly better results than those of placebo in CFF. However, in the present study using eszopiclone, CFF at 4:00 hour had better results than placebo. Since eszopiclone has a long half-life compered to zolpidem and zaleplon, the CFF test at 4:00 was affected by the drug efficacy (t1/2 eszopiclone = 4.8–5.1 h, t1/2 zolpidem = 1.7–2.3 h, t1/2 zaleplon = 1–1.5 h) [25]. Although CFF results at 4:00 hour were worse in the subjects with eszopiclone, the other objective parameters, such as, TUG, FRT and body sway test, were the same as those with placebo; therefore the risk of falls would not be changed in these two conditions. In addition, the CFF result at 4:00 would be considered to be the main effect of eszopiclone and would contribute to the maintenance of sleep.

Uemura et al. [16] have reported that ultrashort-acting hypnotics zolpidem and triazolam did not affect cognitive motor function the next day for the elderly. This result is consistent with present study except for CFF result. Suda et al. [26] reported that administration of eszopiclone to healthy young people improved their memory function the next day; however, no significant difference was seen in our study with the elderly. This was due to the difference in the original memory functions of the young and the elderly. Although feeling of deep sleep and the number of WASO were not improved, significant shortening of sleep onset latency was observed by the administration of eszopiclone compared to that of placebo. In subjective assessments, there were no significant differences in SSS, alertness, well-being, and fatigue on the following day.

## Limitations

We are aware of several limitations of this study: small sample size, only a short-term observation, and lack of objective assessment of sleep such as PSG or actigraphy. In addition, the examinations were conducted on the healthy elderly only at 4:00 in the early morning, and the influence on insomniacs at midnight was not considered. Therefore, these results were different from those of patients with chronic insomnia and midnight urinations. Further studies of elderly insomniacs with midnight awakenings are needed.

## Conclusion

Based on the above results of the changes of physical and cognitive functions in the healthy elderly after taking hypnotics, it would be recognized that eszopiclone 1mg is likely to be unharmed for the healthy elderly.
